# Outcomes of Meek micrografting versus mesh grafting on deep dermal and full thickness (burn) wounds: Study protocol for an intra-patient randomized controlled trial

**DOI:** 10.1371/journal.pone.0281347

**Published:** 2023-02-14

**Authors:** Danielle Rijpma, Anouk Pijpe, Karel Claes, Henk Hoeksema, Ignace de Decker, Jozef Verbelen, Paul van Zuijlen, Stan Monstrey, Annebeth Meij-de Vries

**Affiliations:** 1 Burn Center, Red Cross Hospital, Beverwijk, The Netherlands; 2 Amsterdam UMC Location Vrije Universiteit Amsterdam, Plastic, Reconstructive and Hand Surgery, Amsterdam, The Netherlands; 3 Association of Dutch Burn Centers, Burn Center, Red Cross Hospital, Beverwijk, The Netherlands; 4 Department of Plastic Surgery, Ghent University Hospital, Ghent, Belgium; 5 Ghent Burn Center, Ghent University Hospital, Ghent, Belgium; 6 Department of Plastic, Reconstructive and Hand Surgery, Red Cross Hospital, Beverwijk, The Netherlands; 7 Department of Pediatric Surgery, Amsterdam UMC, Amsterdam, The Netherlands; 8 Department Surgery, Red Cross Hospital, Beverwijk, The Netherlands; University of California Davis, UNITED STATES

## Abstract

**Introduction:**

Autologous split thickness skin grafting is the standard-of-care for most deep dermal and full thickness skin defects. Historically, mesh grafting is used to expand skin grafts for smaller defects and other techniques such as Meek micrografting is used to enable expansion for larger skin defects. Yet, Meek micrografting is increasingly used for smaller skin defects as well. Both techniques are frequently used, especially in burn centers, but evidence on which one is preferable for relative smaller skin defects is lacking. Therefore, an intra-patient randomized controlled trial was designed to adequately compare multiple outcomes of the Meek micrografting and mesh grafting techniques.

**Materials and methods:**

A multicenter intra-patient controlled randomized trial is being performed in two burn centers (the Netherlands and Belgium) to compare multiple outcomes of Meek micrografting and mesh grafting burns or skin defects. Study registration number (NL74274.029.20). Adult patients with a (burn) wound and an indication for surgical excision and skin grafting were screened for inclusion. In total 70 patients will be included and the primary outcome is scar quality twelve months post-surgery assessed by the Patient and Observer Scar Assessment Scale. Moreover, graft take, re-epithelialization, infection rate, donor site size and patients’ preference are also measured within hospital admission, on 3 months and 12 months post-surgery.

**Discussion:**

This is the first randomized trial that is intra-patient controlled, which enables a proper comparison between both skin expansion techniques. The results of this study will contribute to the clarification of the indications of both techniques and ample attention is paid for the patients’ opinion on the surgical treatment options.

## Introduction

Surgical debridement followed by autologous skin grafting is standard-of-care in the treatment of most deep dermal or full thickness (burn) wounds, and split thickness skin grafting (STSG) is one of the most commonly used methods. Skin grafts are often fenestrated, allowing graft expansion and a reduction of the size of donor sites (unaffected skin used to harvest skin grafts). These fenestrations also permit drainage of potential hematoma or seroma [1].

Mesh skin grafting, a technique first described in 1964 by Tanner et al., is one of the most widely used expansion techniques due to its quick and easy application [2]. Although meshed grafts can theoretically be expanded up to 9 times the initial surface area, expansion rates of 1:4 and greater becomes less manageable for surgeons and can lead to re-epithelization delay, wound desiccation and to a more evident “*string-vest*” appearance [3–5]. Consequently, this technique is the most suitable for (burn) wounds up to a maximum of 20% total body surface area (TBSA). In larger (burn) wounds mesh grafting is possible but less favorable [4]. These wounds require large skin graft expansion rates to achieve adequate wound coverage. Meek micrografting, first described in 1958 by Dr. Cicero Parker Meek in the USA, is an alternative skin expansion technique that allows actual expansion rates up to 1:9 has proven effective in coverage of extensive (burn) wounds with limited donor site areas [4, 6, 7]. Consequently, it is used in particular for (burn) wounds larger than 20% TBSA [7]. Yet, Meek micrografting is increasingly used for smaller (burn) wounds, as illustrated by the development of Meek plissés with an expansion ratio of 1:2.

While both expansion techniques are being used in specialized burn centers worldwide, consensus on which technique is preferable for smaller (burn) wounds (TBSA <20%) is lacking. There is a shortage of studies comparing the (long-term) outcomes of both techniques [7]. Studies regarding scar quality are particularly lacking, though this outcome parameter has a great influence on the patients’ social, psychological and/or functional rehabilitation [8]. In addition, Meek micrografting appears in our clinical experience to result in a good scar quality and smaller donor sites due to larger expansion.

We therefore designed an intra-patient randomized controlled trial to compare the outcomes of the Meek micrografting technique and the mesh skin grafting technique on deep dermal and full thickness (burn) wounds up to 20% TBSA. The scar quality on 12 months post-surgery is our primary outcome parameter.

## Materials and methods

### Study design

This is a multicenter, intra-patient, randomized, controlled trial, comparing the Meek micrografting and mesh grafting technique. The study is being performed in the burn centers of the Red Cross Hospital Beverwijk in the Netherlands and the University Hospital Ghent in Belgium between 2021–2024.

### Protocol and registration

**Fig 1:**
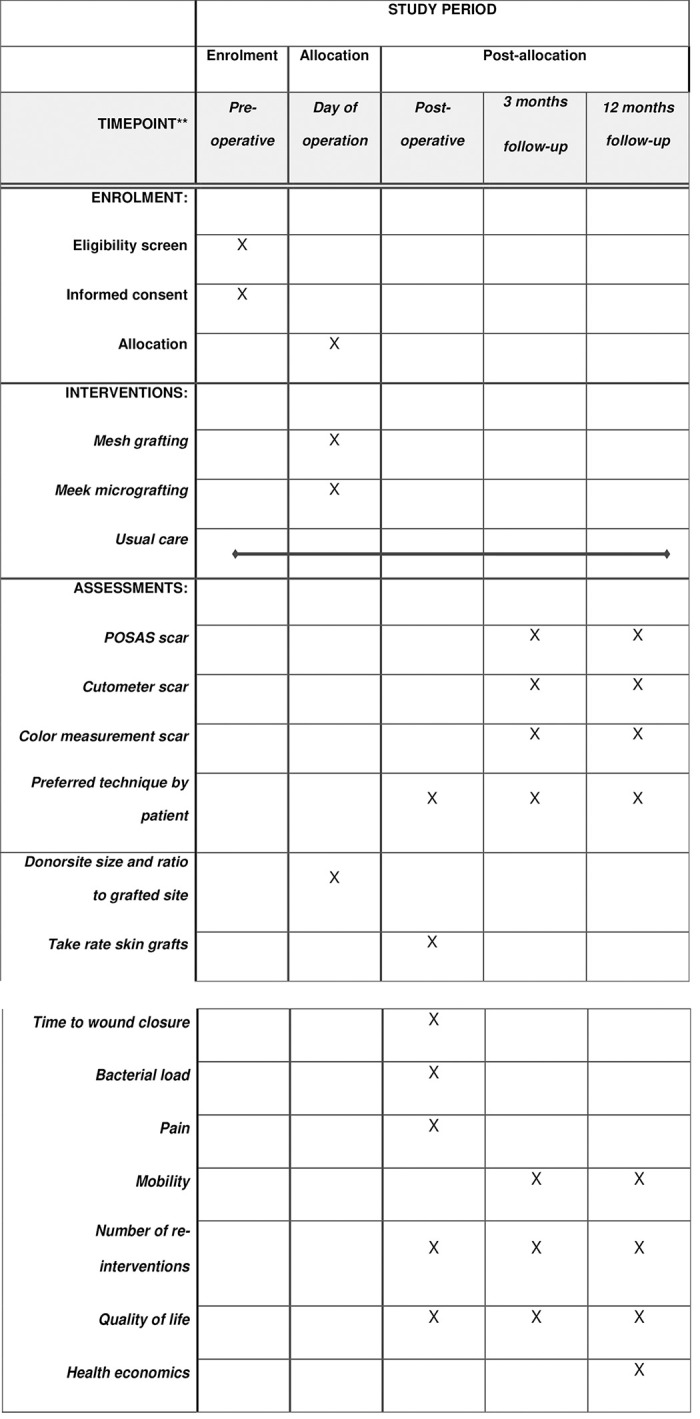
SPIRIT figure: Schedule of enrollments, interventions and assessments.

The study is being conducted according to the principles of the Declaration of Helsinki (64th WMA General Assembly, Fortaleza, Brazil, October 2013), General Data Protection Regulation and Good Clinical Practice. In the Netherlands, approval of this study was given by the Medical Ethics Committee of the VU medical center (NL74274.029.20) and the Review Board of the Red Cross Hospital in the Netherlands. Belgian permission was given by the Medical Ethical Committee and the Review Board both of the University Hospital Ghent (B670201942116). The study methods are registered at the https://trialsearch.who.int/ (NL8847) and the Central Committee on Research Involving Human Subjects (NL74274.029.20) and documented in the protocol. The protocol is designed in accordance with the SPIRIT (Standard Protocol Items: Recommendations for Interventional Trials). See Fig 1 for the SPIRIT Figure and S1 Appendix. for the SPIRT Checklist. After study completion, all underlying study data will be made available. There are no restrictions on publication of study data.

### Participants

All patients 18 years and older admitted to one of the two burn centers with (burn) wounds with an indication for surgical debridement and skin grafting are screened for enrollment in this study. See Table 1. for an overview of in- and exclusion criteria. Moreover, wounds that could be covered with a full sheet, mesh 1:1 or mesh 1:1.5 STSG are not included. Eligible patients or their legal representative receive both oral and written information concerning this clinical trial from one of the members of the research team and have at least 24 hours to decide whether they want to participate in this trial. In case of participation, written informed consent is obtained.

**Table 1:** In- and exclusion criteria for enrollment in this study.

Inclusion criteria	Exclusion criteria
Patients ≥18 years	Patients who participated in another study utilizing an investigational drug or device within the previous 30 days
Patients with: • two comparable deep partial thickness and/or full thickness (parts of) burns or skin defects • in case of burns, confirmed with laser Doppler whenever possible • minimum 36 cm^2^ (= one 1:2 Meek plissé) per study area - requiring surgery after assessment by a (plastic) surgeon/burn physician	Patients with wounds only covering face, hands or joints
	Patients who has one or more medical condition(s) that in the opinion of the treating physician would make the patient an inappropriate candidate for this study
Patients who are mentally capable to give legal consent or when the patient is temporarily incompetent (e.g. patient is sedated/ventilated), a legal representative who can give legal consent	Patients who are expected (according to the responsible medical doctor) to be non-compliant to the study protocol. (This included patients with severe cognitive dysfunction/impairment and severe psychiatric disorders).

## Interventions

### Mesh graft technique

First, wound debridement is performed and hemostasis is secured. All wound debridement techniques (for instance; hand held knifes, hydrosurgery and/or enzymatic debridement) may be used. Thereafter, a split thickness skin graft is harvested with a Zimmer dermatome (Zimmer Biomet Foundation Inc., Warsaw, USA) from the donor site area. The required STSG size is extensively calculated in advance to diminish excessive grafting. However, when a small piece of STSG is excessively harvested, this is noted in the patient’s case report form (CRF). After harvesting, adrenaline soaked gauzes are temporary placed on the donor site. Subsequently, a foam wound dressing is placed on the donor site, and the skin graft is meshed with the corresponding carrier of the selected expansion rate. Skin expansion is enabled by the multiple small perforations in the STSG made by the meshing machine. The meshed graft is placed and trimmed on the wound and fixated with Urgotul^®^/Surfasoft^®^ and staplers. Finally, the meshed grafts is covered with gauzes drenched in antimicrobial solutions based on wound cultures and topped with dry sterile gauzes and bandages.

### Meek micrograft technique

**Fig 2:**
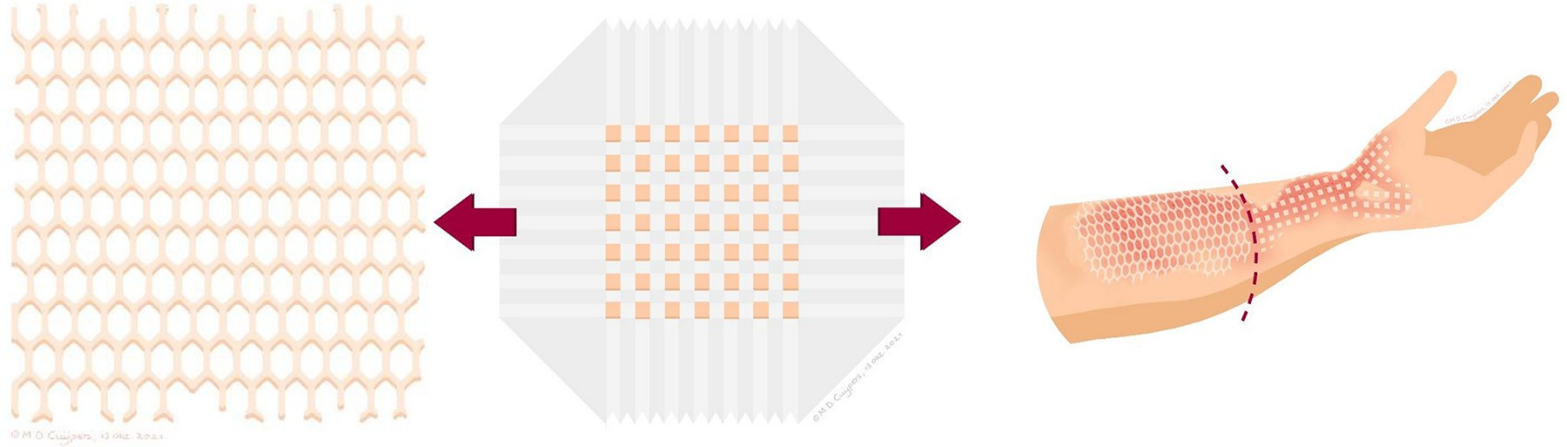
a. Illustration of a mesh graft. b. Illustration of Meek micrograft on a plissé. c. Illustration of a wound divided into two study areas, one covered with a mesh graft and one with a Meek micrograft.

Debridement of the wound, harvesting of the STSG and wound care of the donor site area are performed similarly to the mesh grafting procedure. The required STSG is preferably harvested subsequent to the donor site of the previous harvested STSG for the other technique (one contiguous donor site). Another option is to harvest a separate STSG next to the previous harvested STSG (two separate donor sites). The STSG is displayed with the dermal site on a cork square (42x42 mm) and will be cut in 196 squares by a Meek micrograft cutting machine. Next, the sliced graft on cork is sprayed with special glue and placed on a pre-folded dual gauze, a plissé, which determines the expansion rate. After removal of the cork, the skin expansion is obtained by traction on all four sides of the plissé. The firm supportive layer of the plissé is removed, leaving a single layered gauze with 196 separated graft squares. Finally, the plissé with the graft squares is applied to the wound and fixated with staples. Similar to the mesh grafting technique, all pre-folded gauzes are covered with gauzes drenched in antimicrobial solutions based on wound cultures and topped with dry sterile gauzes and bandages (Fig 2).

### Randomization and surgical procedures

Prior to surgery, two study areas (study areas A and B) on one patient are pre-defined by the researcher in consultation with the operating team. The surface area of each study area must be at least 36 cm^2^, and the two study areas should correspond regarding the depth, location and wound size. Two separate wound areas are preferably chosen, or alternatively, one wound is divided into two areas. At the start of surgery two wounds swabs are taken from the pre-defined study areas A and B. Thereafter, debridement and hemostasis are performed on both study areas. At this point, comparability of the pre-defined study areas A and B are assessed for the last time and the study areas A and B are definitely defined. Next, the two grafting techniques are randomly allocated to study areas A and B through the Redcap randomization module (REDCap 11.1.19 –© 2022 Vanderbilt University). Study area A is grafted first and expansion rates are equal for both study areas. In general, an expansion ratio of 1:2 is used for wounds < 11% TBSA requiring skin grafting and an expansion ratio of 1:3 is used for wounds between 11 and 20% TBSA requiring skin grafting. Wound dressings and treatment pre- and post-surgery are performed according to local standard-of-care. Both study areas are equally treated, namely for the immediate postoperative period gauzes with antimicrobial solutions based on wound cultures. If this results in a different treatment within the study areas, this is noted in the CRF.

### Blinding

Given that patients are under general anesthesia during surgery, they are unaware which grafting technique is used on the study areas so are blinded. During surgery it is not possible to blind the operating team, since they are aware which wound receives Meek micrografting or mesh grafting. Outcome assessment is made as blinded as possible, since the investigator doing the follow-up measurements is unaware of the used techniques in study areas A and B.

### Study outcomes

#### Primary outcome

Scar quality at 12 months post-surgery assessed with the Patient and Observer Scar Assessment Scale (POSAS) 2.0 is the primary study outcome [9, 10]. The POSAS, a subjective measurement tool to quantify scar quality, consists of two numeric scales; the observer scale and the patient scale. Both scales assess scar quality with a 10-point rating system (1 represents normal skin and 10 the worst possible scar) on several items. The observer scale contains an overall scar opinion and 6 separate scar items: vascularity, pigmentation, thickness, relief, pliability and surface area. The patient scar measurements consist of an overall scar opinion and 6 following scar items; pain, itch, color, stiffness, thickness and irregularity. Both study areas A and B are evaluated separately by the patient and two trained observers, independent from each other.

#### Secondary outcomes

**Fig 3:**
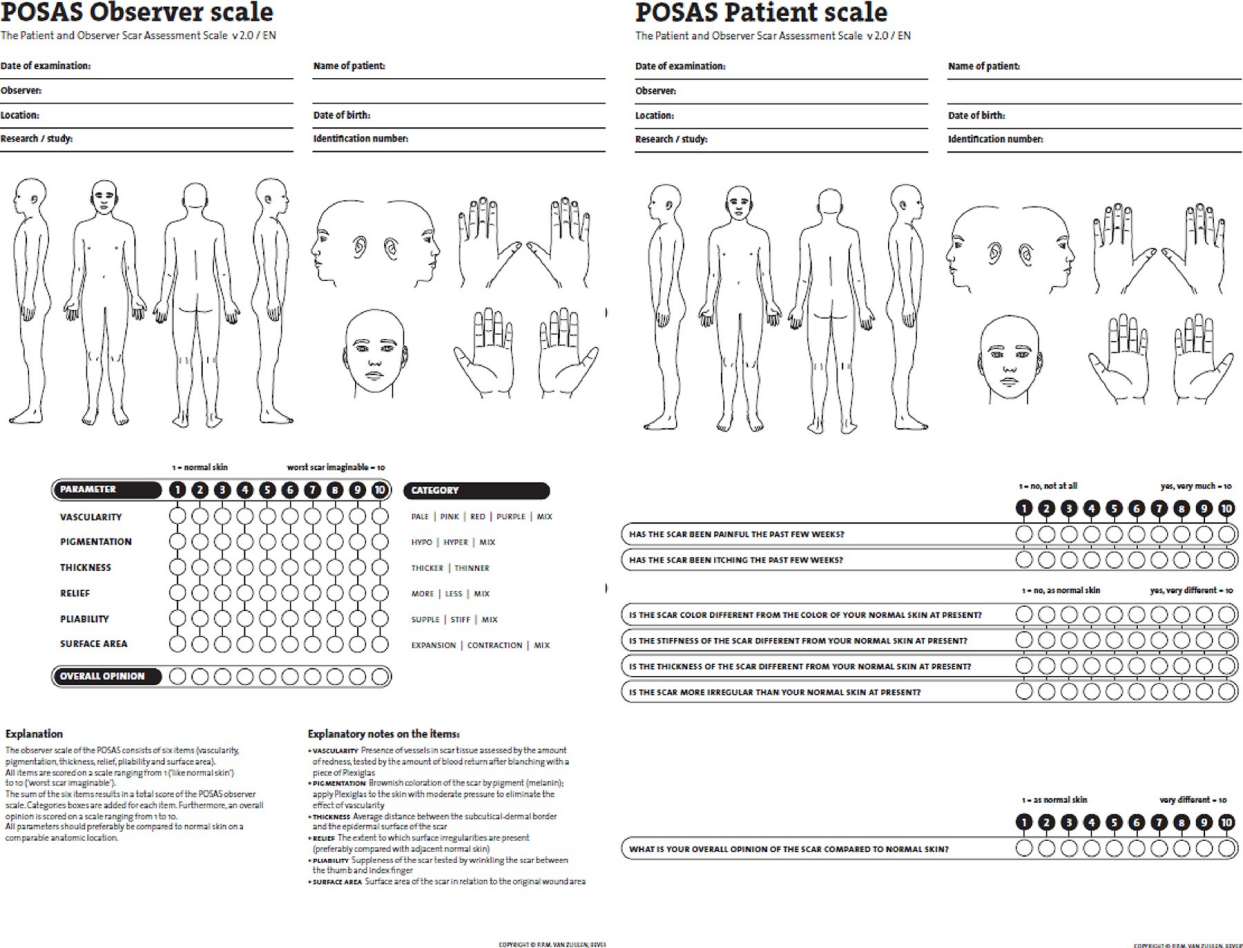
a. Observer scale op de Patient and Observer Scar Assessment Scale (POSAS) 2.0. b. Patient scale op de Patient and Observer Scar Assessment Scale (POSAS) 2.0.

*Scar quality*. Scar quality is measured 3 and 12 months post-surgery with both subjective and objective tools. The POSAS 2.0 is used for subjective scar quality measurement (Fig 3). Objective scar quality measurements include assessment of scar color and pigmentation with the Mexameter (Courage + Khazaka Electronic GmbH, Cologne, Germany) or Dermaspectrometer (CORTEX Technology, Hadsund, Denmark). The erythema- and melanin index are absolute values that represent the disparity in vascularization and pigmentation between a study area and unharmed skin. In each study area, five measurements of the erythema- and melanin index are performed. One control measurement is conducted on unharmed skin at a similar location as the assessed scar [11]. The Cutometer (MPA 580, Courage + Khazaka electronic GmbH) is used to assess skin elasticity (Ue), extension (Uf), pliability (Ua), retraction (Ur) and viscoelasticity (Uv), which will be expressed in percentages. The Cutometer creates a vacuum that pulls the skin towards the probe, these skin movements are measured in millimeters. In each study area five measurements are completed and one control measurement is performed on a comparable location of unharmed skin [12]. Scar treatment is based on the patient’s complaints such as hydration, pressure garments, silicon therapy, laser therapy and reconstructive surgery. These interventions are noted in the patient’s CRF until the final follow-up appointment 12 months post-surgery. It is intended to treat both study areas equally, when this is not possible it is noted in the patient’s CRF.

*Preferred skin graft technique by patient*. Patients are asked: ‘if you would have a new (burn) wound which requires skin grafting; which technique would you prefer?’. Three answer options are given: A, B, or no preference. The patient’s satisfaction level for both techniques is assessed with a self-compiled short survey consisting of two questions regarding the satisfaction for study areas A and B on a 5-point Likert scale. All these questions are asked weekly during admission and at the follow-up moments of 3 and 12 months post-surgery.

*Donor site size and ratio of donor site size and actual graft size*. Surface areas of the donor sites and the study areas A and B are calculated with a 3D-camera (inSight 3D wound measurement, eKare, Fairfax, USA). This provides information of the required donor site size estimation and on the actual graft expansion of Meek micrografting and mesh grafting.

*Take rate of skin grafts*. Assessment of the graft take is performed at 8 +/- 2 days post-surgery by a burn physician / (plastic) surgeon and is expressed as the estimated percentage taken of the applied Meek micrograft or mesh graft.

*Time to complete wound closure*. Time to complete wound closure is defined as the number of days post-surgery at which the wound is re-epithelialized for >95%, has no more drainage and does no longer need a substantial wound dressing. This is assessed at 14 and 21 days +/- 2 post-surgery by a burn physician / (plastic) surgeon.

*Bacterial load*. Wound swabs are taken from study areas A and B in the operation room prior to debridement and at the first wound inspection on 8+/-2 days post-surgery. These are used for semi-quantitative bacteriology analysis in both groups. For both study areas the percentage of clinical wound infections requiring systemic antimicrobial therapy are registered. ‘Clinical infection’ is defined as the presence of cellulitis and/or visible purulence and/or lymphangitis combined with one or more of the following: local wound pain, erythema, edema or malodor.

*Pain*. Pain scores are evaluated for both study areas with the Visual Analogue Scale (VAS) 10-point scale. Assessment takes place before and after removal of gauzes on day 1 post-surgery, and thereafter weekly during admission. After hospital discharge, pain is be evaluated again at 3 and 12 months follow-up.

*Number of secondary procedures*. The amount of re-interventions in study areas due to insufficient graft take or necessity of reconstructive surgery are registered up to 12 months post-surgery.

*Mobility*. Quick DASH questionnaire and the Lower Extremity Functional Scale (LEFS) are used if both study areas are located on the upper extremities or the lower extremities respectively [13, 14].

*Quality of Life (QoL)*. Health related quality of life are assessed with three questionnaires: EQ5D-5L, SF36 and DLQI [15–17]. Questionnaires are completed at hospital discharge, at 3, and 12 months post-surgery. Patients will complete each questionnaire twice, taking into account study areas A and area B.

*Health economics*. An elaborate health economic model comparing Meek micrografting and mesh grafting skin expansion techniques will be constructed with the incorporation of both short-term and long-term costs based on subjective and objective financial data. Objective financial data will be included but is not limited to costs per day, hospitalization time, used materials, operating time and number of corrective surgical procedures. Subjective financial data will be based on the various subjective scales (POSAS, SF-36, EQ5D-5L), recorded for both procedures intra-individually at every moment of follow-up. The subjective data will be used in the development of a cost-utility model, accounting for the difference in cost per quality-adjusted life years (QALY) for both techniques. An assessment of incremental QALY’s will enable widespread comparability given that it is an official unit of measurement in the field of health economics. Using regression analysis on the previously mentioned variables it will be feasible to construct a representative economic model.

*Incidence of AE and SAE*. There are no Adverse Events (AEs) expected, relating to this study (comparison of two already practiced skin grafting techniques). A Serious Adverse Event (SAE) is any untoward medical occurrence in a subject who is participating in a clinical study performed. This is defined as an event that is: a) fatal, b) life-threatening, c) requires or prolongs inpatient (unexpected) hospitalization, these SAEs will be reported directly to the Medical Ethics Committee. SAEs related to surgical treatment in general will be noted in a line-listing and will be reported yearly to the Medical Ethics committee. These are defined as an event that results in a: a) pneumonia, b) urinary tract infections, c) sepsis.

#### Sample size

A Paired t-Test for Mean Difference was used to perform a sample size calculation based on scar quality as expressed by POSAS at 12 months post procedure. For a superiority trial comparing 2 paired means, a sample size of 63 patients yields 90% power when assuming a true mean difference of 1, a standard deviation of 2.4 and a moderate correlation of 0.5. The power calculation is based on a prospective observational study on outcome after burns. The minimally important clinical difference of the POSAS score is unknown, the true mean difference of 1 is therefore based on our clinical experience [18]. Expecting a dropout rate of 10%, the sample size is increased to 70 patients.

#### Statistical analysis

SPSS PASW Statistics 25.0 (IBM, New York City, NY, USA) [19] will be used to perform data analysis. Potential differences in all outcome parameters will be assessed. First, for all outcome parameters, descriptive statistics and univariable comparisons, by the means of a paired *t-*test or Wilcoxon signed-rank test depending on distribution will be performed. Second, for the longitudinal assessed scar quality outcome parameters such as POSAS, scar color, scar elasticity, preference patient, quality of life and mobility surveys we will use a multivariable mixed model analysis considering the different POSAS items, time points and possible effect modificators and/or confounders including patient and burn characteristics like burn depth, age or sex.

## Discussion

We have described the protocol for our multicenter intra-patient randomized controlled trial that compares outcomes of the Meek micrografting and mesh grafting techniques on deep dermal and full thickness (burn) wounds. The potential difference between scar quality of both techniques measured with the POSAS at 12 months is the primary outcome. Donor site size and patient preferences are important secondary outcomes.

To the best of our knowledge, this is the first multicenter intra-patient randomized controlled study that compares outcomes of the Meek micrografting and mesh grafting technique on (burn) wounds. Our research team conducted a review to the outcomes of the Meek micrografting technique, which showed the quality of studies on this skin expansion technique is poor [7]. There is a lack of data on (long-term) outcomes of Meek micrografting and of the comparison between outcomes of the Meek micrografting and mesh grafting techniques. The development of Meek plissés with an expansion ratio of 1:2 illustrates that the indication for Meek micrografting is broadening to smaller wounds. In addition, Meek micrografting appears to provide promising scar quality results in the clinical setting. Therefore, this is the opportune moment to perform a comparative study to the outcomes of the Meek micrografting and mesh grafting techniques.

This study has several strengths. First, the broad inclusion criteria of this study will provide a diverse sample size, which will correspond as much as possible with the actual patient population of our burn centers. Second, comparison of both skin expansion techniques in the same patient will lead to less bias regarding the patient specific processes, such as wound healing and scar formation. In addition, the POSAS is a validated scar assessment scale that includes evaluations of both the clinician (observer) and the patient, and is worldwide the most frequently used patient-reported scar quality assessment scale [9, 20]. Patient-rated scar evaluation scales such as the POSAS are known to be directly linked to the patient’s psychological distress [8, 21]. In combination with multiple secondary outcome parameters on both short- and long-term, a cost analysis and the patients’ preference of both techniques, this study will provide a comprehensive assessment of Meek micrografting and mesh grafting for (burn) wounds.

There is also a limitation of this study. Physicians cannot be blinded for the skin expansion techniques, since they perform the skin grafting procedures. In addition, one of the researchers is aware of the randomization results and therefore is not blinded. Finally, the patterns of the Meek micrograft and mesh grafts are known by both physicians and researchers and could remain visible during woundhealing and scar formation. This makes complete blinding within a study assessing Meek micrografting and mesh grafting impossible. Still, we are trying to perform our study procedures as blinded as possible by indicating the transplanted wounds by study areas A and B, instead of using the terms Meek micrograft and mesh graft. Besides, outcome assessment is made as objective as possible, since the investigator doing the follow-up measurements is unaware of the used techniques in study areas A and B.

The aim of this study is to provide evidence on the pros and cons of Meek micrografting and mesh grafting on relative smaller (<20% TBSA) wounds. Considering these into, the physician and patient could discuss and decide together on which of the two skin expansion techniques would obtain the best outcome.

## Supporting information

S1 Appendix SPIRIT checklist. (PDF)

S2 Appendix Study protocol MvsM. (PDF)

## Abbreviations

STSG: split thickness skin graft
TBSA: total body surface area
POSAS: Patient and Observer Scar Assessment Scale
SPIRIT: Standard Protocol Items: Recommendations for Interventional Trials
