# Supplementary material for: Outcomes of Meek micrografting versus mesh grafting on deep dermal and full thickness (burn) wounds: Study protocol for an intra-patient randomized controlled trial
Source: PLoS One. 2023 Feb 14;18(2):e0281347. doi: 10.1371/journal.pone.0281347 (PMC9928116; doi:10.1371/journal.pone.0281347)
Supplement: S2 Appendix — (PDF) [file pone.0281347.s002.pdf]

RESEARCH PROTOCOL

A multicenter randomized controlled trial assessing and comparing long-term scar quality after micrografting versus mesh grafting of deep dermal burns

Version 3.0, 17 December 2020

PROTOCOL TITLE ‘A multicentre randomized controlled trial assessing and comparing long-term scar quality after micrografting versus mesh grafting of deep dermal burns’

**Protocol ID**	NL74274.029.20
**Short title**	*Meek vs Mesh*
**EudraCT number**	*Not applicable*
**Version**	*2.0*
**Date**	*29 October 2020*
**Coordinating investigator/project leader**	*Drs. D. Rijpma, MD, PhD candidate*<br>*Red Cross Hospital*<br>*1940 EB Beverwijk*<br>*Postbus 1074*<br>*drijpma@rkz.nl*
**Principal investigator(s) (in Dutch: hoofdonderzoeker/ uitvoerder)**<br>***<Multicenter research: per site>***	***Ghent:***<br>*Prof. dr. S. Monstrey, MD, PhD*<br>*University Hospital Ghent*<br>*9000 Ghent, Belgium*<br>Stan.Monstrey@UGent.be<br><br>***Beverwijk:***<br>*Dr. A. de Vries, MD, PhD*<br>*Red Cross Hospital*<br>*1940 EB Beverwijk*<br>*Postbus 1074*<br>adevries@rkz.nl
**Sponsor**	*Ghent University Hospital, Belgium*
**Subsidising party**	*FWO/TBM Grant (2019-2024) no: T000319N*
**Independent expert (s)**	*Dr. E. de Jong, MD, PhD*<br>*Red Cross Hospital*

	Vondellaan 13<br>*1940 EB Beverwijk*<br>*Postbus 1074*
**Laboratory sites**	*Not applicable*
**Pharmacy**	*Not applicable*

PROTOCOL SIGNATURE SHEET

**Name**	**Signature**	**Date**
**Sponsor representative**<br>*Prof. dr. S. Monstrey, MD, PhD*<br>*University Hospital Ghent, Belgium*		29-10-2020
**Principal investigators**<br>*Dr. A. de Vries, MD, PhD*<br>*Red Cross Hospital, Beverwijk, the Netherlands*		29-10-2020

TABLE OF CONTENTS

1. INTRODUCTION AND RATIONALE 11
2. OBJECTIVES 13
3. STUDY DESIGN 14
4. STUDY POPULATION 15
4.1 Population (base) 15
4.2 Inclusion criteria 15
4.3 Exclusion criteria 15
4.4 Sample size calculation 15
5. TREATMENT OF SUBJECTS 17
5.1 Investigational product/treatment 17
5.2 Use of co-intervention (if applicable) 17
5.3 Escape medication (if applicable) 17
6. INVESTIGATIONAL PRODUCT 18
6.1 Name and description of investigational product(s) 18
6.2 Summary of findings from non-clinical studies 18
6.3 Summary of findings from clinical studies 18
6.4 Summary of known and potential risks and benefits 19
6.5 Description and justification of route of administration and dosage 20
6.6 Dosages, dosage modifications and method of administration 20
6.7 Preparation and labelling of Investigational Medicinal Product 20
6.8 Drug accountability 20
7. NON-INVESTIGATIONAL PRODUCT 21
7.1 Name and description of non-investigational product(s) 21
7.2 Summary of findings from non-clinical studies 21
7.3 Summary of findings from clinical studies 21
7.4 Summary of known and potential risks and benefits 21
7.5 Description and justification of route of administration and dosage 21
7.6 Dosages, dosage modifications and method of administration 21
7.7 Preparation and labelling of Non Investigational Medicinal Product 21
7.8 Drug accountability 21
8. METHODS 22
8.1 Study parameters/endpoints 22
8.1.1 Main study parameter/endpoint 22
8.1.2 Secondary study parameters/endpoints (if applicable) 22
8.1.3 Other study parameters (if applicable) 24
8.2 Randomisation, blinding and treatment allocation 24
8.3 Study procedures 24
8.4 Withdrawal of individual subjects 26
8.4.1 Specific criteria for withdrawal (if applicable) 26
8.5 Replacement of individual subjects after withdrawal 27
8.6 Follow-up of subjects withdrawn from treatment 27

8.7 Premature termination of the study....27
9. SAFETY REPORTING....28
9.1 Temporary halt for reasons of subject safety....28
9.2 AEs, SAEs and SUSARs....28
9.2.1 Adverse events (AEs)....28
9.2.2 Serious adverse events (SAEs)....28
9.2.3 Suspected unexpected serious adverse reactions (SUSARs)....29
9.3 Annual safety report....30
9.4 Follow-up of adverse events....30
9.5 [Data Safety Monitoring Board (DSMB) / Safety Committee]....30
10. STATISTICAL ANALYSIS....31
10.1 Primary study parameter(s)....31
10.2 Secondary study parameter(s)....31
10.3 Other study parameters....31
10.4 Interim analysis (if applicable)....31
11. ETHICAL CONSIDERATIONS....32
11.1 Regulation statement....32
11.2 Recruitment and consent....32
11.3 Objection by minors or incapacitated subjects (if applicable)....32
11.4 Benefits and risks assessment, group relatedness....32
11.5 Compensation for injury....32
11.6 Incentives (if applicable)....33
12. ADMINISTRATIVE ASPECTS, MONITORING AND PUBLICATION....34
12.1 Handling and storage of data and documents....34
12.2 Monitoring and Quality Assurance....34
12.3 Amendments....34
12.4 Annual progress report....34
12.5 End of study report....35
12.6 Public disclosure and publication policy....35
13. STRUCTURED RISK ANALYSIS....36
13.1 Potential issues of concern....36
13.2 Synthesis....36
14. REFERENCES....36

LIST OF ABBREVIATIONS AND RELEVANT DEFINITIONS

ABR	General Assessment and Registration form (ABR form), the application form that is required for submission to the accredited Ethics Committee; in Dutch: Algemeen Beoordelings- en Registratieformulier (ABR-formulier)
AE	Adverse Event
AR	Adverse Reaction
CA	Competent Authority
CCMO	Central Committee on Research Involving Human Subjects; in Dutch: Centrale Commissie Mensgebonden Onderzoek
CV	Curriculum Vitae
DSMB	Data Safety Monitoring Board
EU	European Union
EudraCT	European drug regulatory affairs Clinical Trials
GCP	Good Clinical Practice
GDPR	General Data Protection Regulation; in Dutch: Algemene Verordening Gegevensbescherming (AVG)
IB	Investigator's Brochure
IC	Informed Consent
IMP	Investigational Medicinal Product
IMPD	Investigational Medicinal Product Dossier
METC	Medical research ethics committee (MREC); in Dutch: medisch-ethische toetsingscommissie (METC)
POSAS	Patient and Observer Scar Assessment Scale
(S)AE	(Serious) Adverse Event
SPC	Summary of Product Characteristics; in Dutch: officiële productinformatie IB1-tekst
Sponsor	The sponsor is the party that commissions the organisation or performance of the research, for example a pharmaceutical company, academic hospital, scientific organisation or investigator. A party that provides funding for a study but does not commission it is not regarded as the sponsor, but referred to as a subsidising party.
SSG	Split thickness skin graft
SUSAR	Suspected Unexpected Serious Adverse Reaction
TBSA	Total Burned Surface Area
UAVG	Dutch Act on Implementation of the General Data Protection Regulation; in Dutch: Uitvoeringswet AVG

WMO **Medical Research Involving Human Subjects Act; in Dutch: Wet Medisch-wetenschappelijk Onderzoek met Mensen**

SUMMARY

Rationale: Deep dermal / sub dermal (burn) wounds need surgical excision followed by skin grafting to achieve rapid wound healing. Most common method is split thickness skin grafting (SSG). However, in extended (burn) wounds there is a lack of donorsite to harvest skin transplants Moreover, regarding donorsite morbidity it is always high priority to keep donorsite as small as possible. For these reasons, skin grafts are often expanded before application on the wound. Skin graft expansion is generally performed by meshed skin grafting technique, seen this method is considered to be quick and easily applicable. Yet, this technique also has several limitations. For the surgeon this method becomes more cumbersome when the expansion ratio increases. Moreover, the actual expansion of the skin graft is usually lower than the intended expansion ratio and the "fish-net" pattern often stays visible in the eventual scar. An alternative for skin graft expansion is the micrografting technique (Meek technique). In comparison with meshed skin grafting this technique is able to reach true large expansion ratios and thereby maintain small donorsites. As a consequence, this technique is used in particular for very extended (burn) wounds. Both expansion methods are used worldwide in specialized burn centers. Wound healing seems to be similar according to previous published literature. Experience shows a possible advantage of micrografting on scar quality. Three studies compared these two expansion techniques, however none primarily investigated possible differences within long-term scar quality in a prospective study setting. To compare long-term scar quality of micro- versus mesh grafting techniques in deep/subdermal (burn) wounds and thereby providing evidence for the use of micrografting not only for extensive but also for smaller (burn) wounds a randomized controlled trial is highly preferred.

Objective: To assess long-term scar quality and donorsite size of deep dermal or sub dermal (burn) wounds after skin grafting with micrografting compared to mesh grafting technique.

Study design: Multicenter randomized intra-patient controlled trial.

Study population: All adult patients with (burn) wounds affecting 4 - 20% total body surface area with an indication for surgical excision and skin grafting, admitted to the burn center at the Red Cross Hospital in Beverwijk, Netherlands or the burn center of the University Hospital Ghent, Belgium.

Intervention: Prior to surgery two comparable (burn) wounds or two equal parts in one (burn) with a minimum size of 36 cm2 will be selected. These wounds will be randomly allocated to the intervention group (micrografting) or the comparison group (meshed skin grafting).

Main study parameters/endpoints: The main study parameter is scar quality 12 months after either skin grafting. Possible differences in scar quality between both techniques observed by means of Patient and Observer Scar Assessment Scale (POSAS).

Nature and extent of the burden and risks associated with participation, benefit and group relatedness: All patients will have to undergo surgery, independent of skin grafting method. Seen both skin grafting techniques are already been considered as standard treatments, there is no additional risk micrografting compared to meshed skin grafting. The only risk concerns a possible subtle asymmetry of the scar due to two different grafting techniques. However, in practice both techniques are also being combined within one patient. Follow-up broadly corresponds with standard follow-up for (burn) wounds. The duration of check-up at outpatient clinic is slightly increased to 60 minutes, due to additional examinations and surveys.

1. INTRODUCTION AND RATIONALE

Patients with deep dermal burns or full thickness wounds always require a surgical reconstruction with skin grafts to restore the integrity of the outer skin layer. Most of the deep burn wounds are covered with split thickness skin grafts (SSG) which is the standard of care (SOC) treatment nowadays. However, in order to achieve rapid wound closure in extensively burned patients, the shortage of available donor areas for skin graft harvesting represents a significant problem. As a result, coverage of the majority of burn wounds or full-thickness skin defects unavoidably requires an expansion of the skin graft before application.

Currently there are two distinct methods for skin graft expansion. The most frequently used technique worldwide is the easy and fast meshed graft technique whereby the skin theoretically can be enlarged 1.5 to 9 times. The larger the expansion rate, the less donor skin is needed. Unfortunately, it has been demonstrated that in reality the claimed expansion ratios are never achieved with meshed grafts whereby e.g. a 1:3 expansion only results in about 60% increase in size. Therefore, even more donor skin or even larger expansion ratios are needed in order to cover all wounds in severely burned patients. Various additional problems arise due to the use of widely meshed skin grafts, both for the patient and for the surgeon. Mesh-expanded skin grafts take much longer to completely epithelialize and often result in hypertrophic scar formation and contractures. Moreover, in the long-term, a lasting “fishnet or string-vest” pattern will always be visible in meshed grafted areas. An additional technical problem is that skin grafts that are meshed with a ratio above 1:3 are fragile and very difficult to handle for the surgeon.

The second, also currently used method for skin graft expansion is the micrografting technique. It was mainly the need for more manageable and larger expansion ratios in order to increase the survival rate in extensively burned patients that has led to the development of the more labour- and material intensive micrografting technique. With this method, a much more effective expansion of the skin grafts can be achieved, more equal to the claimed expansion ratios resulting in a smaller donor site area. The micrografting technique has been established in many (usually more specialized) burn centers throughout the world due to the easier handling and the lower fragility compared to the widely meshed skin grafts which can be of particular benefit for very extensive burns. For all these reasons, in most burn centers, the micrografting technique is only recommended and reserved for severe burns with at least more than 30-40 % total burned surface area (TBSA).

In addition, and independent of the expansion method, it is important to mention that donor site morbidity actually is an often seriously underestimated problem which in the short term frequently causes the patient more pain than the actual burn.

Since many years, the meshed skin graft technique and the micrografting technique are routinely used (combined) both in Belgium and The Netherlands, therefore providing a true reflection of the current clinical practice. In our burn center extensively (>30%) burned patients are (mostly) treated with the micrografting technique in order to save as much donor site area as possible. During follow-up of many of these patients we often noted a surprisingly good quality of the residual scars following the application of micrografts.

The increased interest in obtaining a good functional and aesthetic outcome after burn surgery is an illustration of the current paradigm shift in burn management from a focus solely on survival towards the overall quality of survival which is often related to the residual scars of the burn patients.

With this in mind and due to the good results obtained in extensively burned patients we started about 6 years ago to use the micrografting technique in much smaller burns. Our clinical experience showed that the initial graft take as well as scar quality were excellent and donorsite morbidity was substantially reduced. These results together with fact that scar quality of meshed graft technique and micrografting technique has never been prospectively to each other, prompted us to compare this within in adequately developed study.

This study involves a large (intra-individual) randomized controlled trial in a multicenter setting to evaluate the effect of two skin grafting techniques both on the quality of scar formation and the reduction of donor site morbidity. This study is crucial in order to provide evidence for a more widespread use of the micrografting technique and this not only for extensively burned patients but also for smaller burns in need of skin graft expansion. All relevant outcome parameters will be evaluated during acute phase and the follow-up period of 12 months.

Our hypothesis is that treatment with micrografting, when compared to conventional meshed skin grafting, will result in a better scar quality and smaller donorsite size.

2. OBJECTIVES

Primary Objective:

- To assess long-term scar quality of deep dermal or sub dermal (burn) wounds after skin grafting with micrografting compared to mesh grafting technique.

Secondary Objectives:

- To assess donorsite morbidity, wound healing, epithelisation, mobility and quality of life after skin grafting with micrografting versus mesh grafting.

3. STUDY DESIGN

This study is a multicenter randomized intra-patient controlled trial conducted in the burn centers of the Red Cross Hospital Beverwijk in the Netherlands and of the University Hospital Ghent in Belgium. Inclusion period is three years. Individual patient follow-up is 12 months.

4. STUDY POPULATION

4.1 Population (base)

All adult patients with (burn) wounds affecting 4 - 20% total body surface area with an indication for surgical excision and skin grafting, admitted to the burncentre at the Red Cross Hospital in Beverwijk in the Netherlands (n=30) or the burncentre of the University Hospital Ghent in Belgium (n=40).

4.2 Inclusion criteria

- Patients ≥18 years
- Clinically deep burn or deep skin defect between 4% and 20% TBSA
- Patients with two comparable deep partial thickness and/or full thickness burns, confirmed by laser Doppler imaging (LDI) or deep skin defects, of minimum 1:2 plissee = 36cm², requiring surgery after assessment by a (plastic) surgeon/burn physician
- Patients who are mentally capable to give legal consent or legal representative when the patient is temporarily incompetent (e.g. patient is sedated/ventilated)

4.3 Exclusion criteria

- Patient has participated in another study utilizing an investigational drug or device within the previous 30 days
- Wounds covering face, hands or joints
- Patient has one or more medical condition(s) that in the opinion of the treating physician would make the patient an inappropriate candidate for this study
- Patients who are expected (according to the responsible medical doctor) to be non-compliant to the study protocol. (This includes patients with severe cognitive dysfunction/impairment and severe psychiatric disorders).

4.4 Sample size calculation

Sample size calculation based on scar quality as expressed by POSAS at 12 months post procedure: Paired t-Test for Mean Difference

Fixed Scenario Elements	
Distribution	Normal
Method	Exact
Number of Sides	2
Alpha	0.05
Mean Difference	1
Standard Deviation	2.4
Correlation	0.5
Null Difference	0

Computed N Pairs			
Index	Nominal Power	Actual Power	N Pairs
1	0.8	0.807	48
2	0.9	0.902	63

For a superiority trial comparing 2 paired means, a sample size of 63 patients yields 90% power when assuming a true mean difference of 1, a standard deviation of 2.4 and a moderate correlation of 0.5. Expecting a drop-out rate of 10% the sample size is increased to 70 patients.

Sample size calculation based on donor site size and expansion rate: Paired t Test for Mean Difference

Fixed Scenario Elements	
Distribution	lognormal
Method	Exact
Number of Sides	2
Alpha	0.05
Coefficient of Variation 1	0.09
Coefficient of Variation 2	0.03
Correlation	0
Nominal power	0.9
Null Geometric Mean Ratio	1

Computed N Pairs

Index	Geo Mean 1	Geo Mean 2	Actual Power	N Pairs
1	1	1.1	0.914	13 (10% difference)
2	1	1.2	0.960	6 (20% difference)

For a paired t test of a lognormal geometric mean ratio with a two-sided significance level of 5%, assuming coefficients of variation of 0.09 and 0.03 (based on Kamolz et al. 2013) and correlation 0 (to be conservative), a sample size of 6 pairs is required to obtain a power of at least 90% to detect a geometric mean ratio of 1.20. (The actual power is 96%.) This number has been increased to 7 patients to allow for a 10% drop-out rate.

Conclusion of sample size calculations:
Taking into account a drop-out rate of 10%, 70 patients will be required to perform this study.

5. TREATMENT OF SUBJECTS

5.1 Investigational treatment

Prior to the first operation the deep/subdermal wounds will be treated according to the local protocol of the burn centre. Subsequently, surgeons will assess if the wound bed is suitable to receive autografts, possibly after additional debridement. When the wounds are eligible for skingraftig, the two wound areas will be defined. Woundswabs and digital photographs will be taken. Randomization will take place before the first surgery and wound areas will be assigned to either micrografting or meshed grafting. For both techniques an expansion rate of 1:2 will be used for a wounds with 4-10% TBSA and 1:3 for wounds with 11-20% TBSA. Donorsite size will be expressed in cm^2 and objectively measured with a 3D camera (Woundworks inSight®). After operation on day 8 +/- 2 days take rate of both micro- and meshed grafts, will be assessed in expressed in percentage of the harvested skin graft.

5.2 Use of co-intervention (if applicable)

Not applicable

5.3 Escape medication (if applicable)

Not applicable

6. INVESTIGATIONAL TREATMENTS

6.1 Name and description of investigational treatments

Micrografting

Skin transplantation by the micrografting technique was first described in 1958 by Cicero Parker Meek in the USA and therefore also referred as the Meek technique. In 1993 the micrografting technique was modified and re-designed by burn surgeon's in the Red Cross Hospital, Beverwijk in association with Humeca Skin Transplantation Technology, Borne, The Netherlands[1]. After harvesting of the autologous skin grafts, the graft will be displayed on a cork square and will be cut in multiple squares by a specialized micrografting cutting machine. Next, the sliced graft on cork will be sprayed with an adhesive layer and will be placed on a pre-folded gauze. This pre-folded gauze expands when all four sides of the gauze are pulled. Finally, the cork will be removed and the gauze with the graft squares are applied to the wound and fixated with staplers. All pre-folded gauzes will be covered with antibacterial gauzes and thereupon dry sterile gauzes and bandages.

Mesh grafting technique

Skin grafts for mesh grafting are harvested by the same method as for the micrografts *(see section 8.3 study procedures for full description of grafting procedures).* After harvesting, the autografts will be displayed over an carrier and pulled through the meshing machine. This cutting machine makes multiple small perforations in the graft, which allows the skin graft to expand. The meshed graft will be attached to the wound and fixated with staplers. Finally, the grafts will be covered with antibacterial gauze and topped with dry sterile gauze and bandages.

6.2 Summary of findings from non-clinical studies

Database collected data from the burncentre of the Red Cross Hospital, Beverwijk, show that within 2 years, 134 patients with burn wounds had an indication for skin grafting. In patients with less extensive burns (0-10% TBSA) the majority, 80% (n=77), received meshed skin grafts. Only 20% (n=18) were transplanted with micrografts. In extensive burns (10-20% TBSA) the amount skin grafting procedures with micrografts increased to 55% (n=22). Whereas the number of meshed skin transplantation reduced to 45% (n=17).

6.3 Summary of findings from clinical studies

Two studies on micrografting versus mesh grafting for patients with burn wounds have been published:

- Zermani et al. (1997)[2] compared functional and aesthetic results after micrografting and after mesh skin grafting. In this prospective study 5 patients with extensive burns (mean TBSA 35%) were included. Expansion ratio from 1:3 to 1:9 were used for micrografts. 6 months post-surgery functional results were similar for both grafting methods. The aesthetic results were better for micrografts. On long-term both functional and aesthetic results were considered good to excellent in all cases. In

comparison with meshed grafts, micrografts enabled skin grafting with a larger expansion ratio and smaller donorsite size.

- Zhen et al. (2019)[3] examined the long-term functional outcomes of micrografting and split skin grafts (SSG) in burns. It's a cross-sectional setting, including burns of 10-40% TBSA at least one year post-burn. 43 patients were included, micrografting n= 15 and SSG n=28. TBSA was significantly higher in the micrografting group, 27% vs. 16% SSG. Functional outcomes were assessed with The Burn Specific Health Score-brief (BSHS-B) and resulted in better scores in micrografting compared with SSG (p>0.05). Vancouver Scar Scale (VSS) was used to evaluate scar quality and showed significantly better scar outcomes after micrografting compared to SSG.

One study was published comparing micrografting with non-micrografting procedures:

- Lumenta et al. (2009)[4] is a retrospective study which among others compared micrografting with non-grafting. They analysed burn severity (%TBSA), age, hospital length of stay, amount of operations and mortality for both groups. Patients in the micrograft group were significantly older, had higher % TBSA but had an equal amount of operations and the same length of hospital stay.

Based on abovementioned studies, micrografting seems to show similar or better results compared with mesh skin grafting especially for extensive burn wounds. However, the evidence for these conclusions is very sparse. A randomized controlled trial is needed to adequately compare the micrografting and mesh graft technique for long-term scar quality. Moreover, since donorsite morbidity is a relevant problem in burn surgery, further development of this micrografting technique is also worthwhile for less extensive (burn) wounds.

6.4 Summary of known and potential risks and benefits

Meshed grafts: This technique for skin expansion and grafting is most widely performed, seen it is a very user-friendly and fast procedure. However, mesh grafting also has its shortcomings. Theoretically an expansion ratio from 1:1.5 until 1:9 are possible[5] In practice, meshed grafts do not expand as much as suggested. Expansion ratios of 1:1,5 and 1:3 turned out to give an surface area expansion of 1,36x and 1,80x respectively[6] Moreover, an expansion ratio above 1:3 is very fragile and therefore difficult to properly cover and fixate on wounds. Besides, the 'fish-net' pattern of the meshed graft remains visible in the long-term scar. For this reason, meshed grafts do not fall within the preferred skin expansion and grafting techniques for extensive wounds with limited donorsite.

Micrografting: True expansion ratios up to 1:9 are achieved, which leads to a smaller donorsite. In comparison with meshed grafts, micrografting will not give fragility problems with a large expansion ratio. Also small graft fragment can be used, in contrast with meshed grafts were only long strips of graft can be utilized (4). Therefore is a preferable technique for especially extensive wounds (2). Graft failure due to bacterial infection is limited to failure of a few islands instead of the whole graft, because of all separate gauzes. Moreover, in our experience the scar quality of mircrografts showed to be very good and might even better

than the scars of meshed grafts. Drawbacks of micrografting are mainly based on a more labour-intensive and time-consuming procedure compared with meshed grafts[7].

6.5 Description and justification of route of administration and dosage

Not applicable.

6.6 Dosages, dosage modifications and method of administration

Not applicable.

6.7 Preparation and labelling of Investigational Medicinal Product

Not applicable.

6.8 Drug accountability

Not applicable.

7. NON-INVESTIGATIONAL PRODUCT/TREATMENT

7.1 Name and description of non-investigational product(s)
Not applicable.

7.2 Summary of findings from non-clinical studies
Not applicable.

7.3 Summary of findings from clinical studies
Not applicable.

7.4 Summary of known and potential risks and benefits
Not applicable.

7.5 Description and justification of route of administration and dosage
Not applicable.

7.6 Dosages, dosage modifications and method of administration
Not applicable.

7.7 Preparation and labelling of Non Investigational Medicinal Product
Not applicable.

7.8 Drug accountability
Not applicable.

Fout! Bladwijzer niet gedefinieerd.

8. METHODS

8.1 Study parameters/endpoints

8.1.1 Main study parameter/endpoint

- Long-term scar quality 12 months after skin transplantation assessed with the Patient and Observer Scar Assessment Scale (POSAS).

8.1.2 Secondary study parameters/endpoints (if applicable)

- Long-term scar quality 3 and 12 months after skin transplantation
  - Subjective measurement of scar quality with Patient and Observer Scar Assessment Scale (POSAS). This is based on multiple aspects of scar formation such as: vascularization, pigmentation, texture, thickness, pliability, itch and pain, and is performed by member of research team and by patient.
  - Objective measurements of scar color and pigmentation with the Mexameter or Dermaspectrometer. Skin elasticity is objectively measured with the Cutometer.
- Skin graft technique preferred by patient
  - Patient gives overall indication as to what skin graft technique he/she would prefer if he/she were to require skin grafting again: weekly during admission, and at 3 and 12 months follow up
- Donorsite size and ratio of donorsite size and actual graft size (cm2)
  - Surface areas will be calculated with a 3D-camera (Woundworks inSight)
- Take rate of skin grafts
  - Take rate will be clinically assessed at 8 +/- 2 days and expressed as the percentage of micrografted and meshed graft take
- Healing time
  - Clinical assessment of wound healing 14 and 21 days +/- 2 post application expressed as percentage of the total wound surface area healed
  - Time to complete wound closure where complete wound closure is defined as > 95% re-epithelialization of the wound with the absence of drainage and no longer needing a substantial wound dressing
- Bacterial load
  - Wound swabs for semi-quantitative investigation
  - Semi-quantitative bacteriology in both groups
  - Percentage of patients within both groups with clinical wound infection requiring systemic antimicrobial therapy
  - ‘Clinical infection’ is defined as the presence of cellulitis and/or visible purulence and/or lymphangitis combined with one or more of the following: local wound pain/erythema/edema/malodor

- Pain
  - Will be evaluated in the autografted target wounds with help of a Visual Analogue Scale (VAS score) on day 2 post surgery, before and after removal of Surfasoft®/Urgotul in case of meshed skin grafts or plissees in case of micrografting, and thereafter once weekly during the hospitalization period. After hospital discharge pain will be evaluated again at the 3 and 12 months follow ups
- Number of secondary procedures
  - Re-interventions in study areas due to insufficient take or necessity of reconstructive surgery
- Mobility at 3 and 12 months
  - If articulations of limbs are involved the Quick Dash for upper extremities and the Lower extremity functional scale for lower extremities, the range of motion will be assessed with a goniometer
- Quality of life
  - Assessed at the time of hospital discharge and at 3 and 12 months after the procedure. 3 quality of life measures, the EQ5D-5L, SF36 and DLQI will be used at different points in time: at the time of hospital discharge and 3 and 12 months post wound healing
- Health economics
  - Differences in operation time, costs of materials/equipment used and staff/personnel and impact of re-interventions and the need for reconstructive surgery will be evaluated. Using regression analysis based on the processing of the EQ5D-5L, SF36 and the DLQI we will be able to construct an economic model. The model will incorporate the short-term costs involved of both skin expansion techniques as well as the long-term costs.
- Incidence of AE and SAE
  - There are no Adverse Events (AEs) expected, relating to this study (comparison of two already practiced skin grafting techniques).
  - A Serious Adverse Event (SAE) is any untoward medical occurrence in a subject who is participating in a clinical study performed. This is defined as an event that is:
    a) fatal
    b) life-threatening
    c) requires or prolongs inpatient (unexpected) hospitalization
  - These SAEs will be reported directly to the METc.
  - SAEs related to surgical treatment in general will be noted in a line-listing and will be reported yearly to the METc. These are defined as an event that results in a:
    a) pneumonia
    b) urinary tract infections
    c) sepsis

d) pulmary embolism

e) re-operation

8.1.3 Other study parameters (if applicable)

- Demographics: age, sex
- Burn characteristics: Percentage total burned body surface area (TBSA), anatomical affected site(s), date of injury, burn depth determined by an experienced burn physician / plastic surgeon and burn depth determined by a LDI-scan on date 2-5 post burn (prior to the operation), date of surgery
- Clinical characteristics: Fitzpatrick skin type, timing to surgery, number of debridements and/or allograft before autografting, comorbidity, use of tangential excision and/or Versajet techniques, dermatome settings, expansion of skin graft

8.2 Randomisation, blinding and treatment allocation

Randomization will be at intra-patient level and performed by means of the Redcap randomization module (most often 5-7 days post allograft application). Preferably 2 wounds in the same patient will be randomized, or alternatively, after discussing this situation with the patient, 1 wound will be divided into 2 halves (A and B). The sequence in which the micrografting and mesh grafting techniques will be applied will also be subject to randomization by means of the Redcap randomization module. In surgical treatment blinding is not possible, as the burn surgeon knows which part of the wound receives what surgical treatment. Outcome assessment will be blinded as the junior investigator/research nurse who does the follow-up measurements is unaware of the technique used in target wound A or B.

8.3 Study procedures

Summary of study procedures

Pre-operatively

- Description of (burn)wounds; cause, cooling, %TBSA, etc.
- Clinical depth assessment by (plastic) surgeon / burn physicians and laser Doppler imaging, in case of burn wounds, between day 2 and 5 post-burn.
- Digital photographs of all (burn) wounds and especially of target wounds.
- Until operation (burn) wounds are treated according to local protocol.

Per-operatively

- Prior to skin grafting debridement(s) and/or allograft applications are performed to prepare wound beds for skin grafting.
- Target wounds will be marked with part A or B with a minimum of 36 cm^2 per wound.
- Digital photographs will be taken of the target wounds.
- Wound swabs for microbiological analysis will be harvested from the wound bed of each target wound before cleaning the wound.
- Randomisation will follow and target wounds will be grafted with the micrografting .technique and the meshed graft technique. Skin grafting techniques are further explained

below (8.3 skin grafting techniques).

<u>Post-operatively and follow-up</u>

- Assessment of study objectives (see section 2 objectives) will be performed during admission in burn centre and several objectives will be assessed at the outpatient clinic 3 and 12 months after operation (see below section 8.3 Follow-up).

Skin grafting techniques

<u>Micrografting</u>

First debridement of the (burn) wounds to be grafted is performed according to local protocol, until a vital wound bed shows. Hemostasis is secured. The surface area of the to be grafted wounds is estimated by the amount of cork squares (42x42 mm) that matches the total wound bed. Thereafter, split thickness skin graft (SSG) of 0.1mm is harvested with the Zimmer dermatome from the in advance selected donorsite area. After harvesting, an alginate wound dressing with adrenaline suspension is placed on the donorsite and the SSG is placed with the dermal site on cork square and cut off. This procedure is repeated until the whole SSG is divided over cork squares, including all small graft fragments. Next, the cork fragments are placed in a holder and passed through the cutting machine. The SSG is cut in 14 long stripes of 3 mm wide. The cork square is now rotated for 90° and passed through the cutting machine again, the graft is now subdivided in little 196 squares of 3x3 mm. Now adhesive spray is applied on the epidermal (upper) site of the graft. When the graft is dried, the cork square is pressed on a double layered pre-folded gauze (plissee). Subsequently, the cork square is removed, which leaves all graft squares on the plissee. Expansion is obtained by traction on all four sides of the plissee, expansion ratio depends on plissee size. The firm supportive layer of the plissee is then removed, leaving a single layered gauze with 196 separated graft squares.

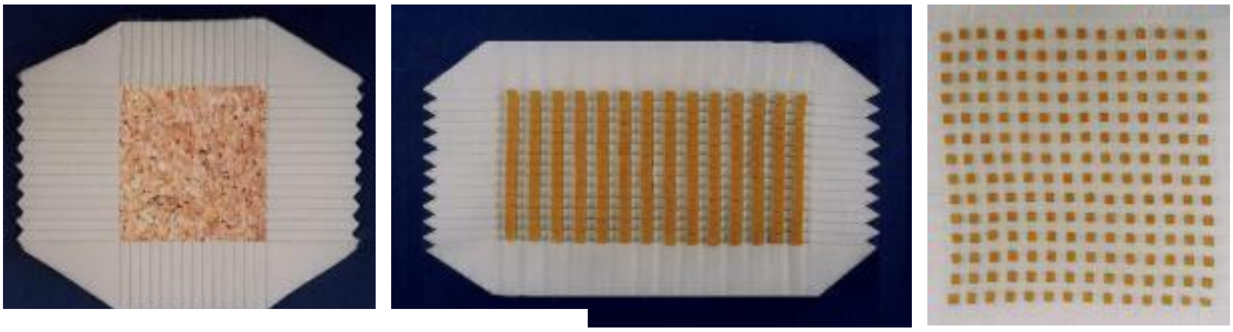

Meek surgery - https://ozmedix.com.au/humeca/meek/

Finally, all single layered plissees are placed with dermal side onto the wound bed and fixated with staplers. Afterwards all ensured plissees are covered with antibacterial gauzes and topped with dry sterile gauzes and bandages. Donorsite is treated with a foam wound dressing with stays on the wound for 14 days. First wound inspection will conducted after at 8 +/- 2 days to assess the take rate of the graft. Hereafter, daily wound dressing changes will be performed. All residual effects will be treated according to the local standard of care.

<u>Meshed grafting</u>

Debridement of the wound bed, harvesting of the SSG and wound care of the donorsite will be performed according to similar procedure as for micrografting. Next, SSG will be placed on a dermacarrier and the meshing machine is set to the preferred expansion ratio. Then the dermacarrier with SSG is passed through the meshing machine. Multiple perforations are made into the SSG, which enables expansion of the graft. Meshed graft will be attached to the wound bed (dermal side down) and fixated with staplers. Finally, the meshed grafts are covered with (antibacterial) gauzes and topped with dry sterile gauzes and bandages.

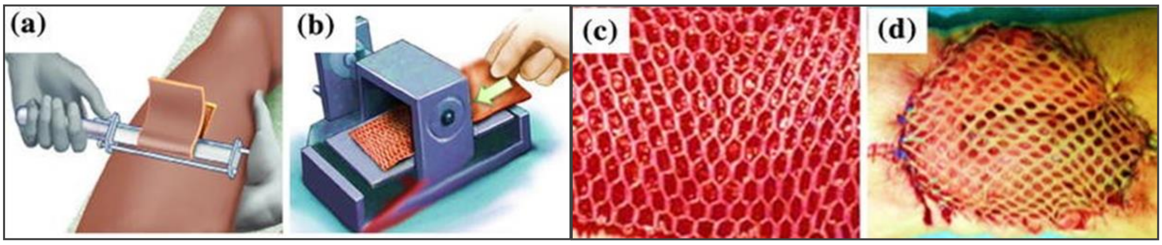

Augustine, R. et al. Advancement of wound care from grafts to bioengineered smart skin substitutes, 2014, Progress in Biomaterials, 3, 103-113

Follow-up

Measurements 3 and 12 months after operation

- Regular check-up after hospital discharge
- Preference skin grafting technique
  - Question preference of patient regarding skin grafting technique on this moment
- Scar quality:
  - Patient and Observer Scar Assessment Scale
  - Scar color and pigmentation measurement with Mexameter or Dermaspectrometer
  - Scar elasticity measurement with Cutometer
- Mobility
  - Questionnaire - Quick Disabilities of the Arm, Shoulder and Hand (Quick DASH) - for (burn)wounds concerning upper extremity
  - Questionnaire - Lower Extremity Functional Scale (LEFS) - for (burn)wounds concerning lower extremity
  - Objective measurement of range of motion with a goniometer
- Quality of life
  - Questionnaire - EQ5D-5L
  - Questionnaire - Short Form Health Survey 36-item (SF36)
  - Questionnaire - Dermatology Life Quality Index (DLQI)

8.4 Withdrawal of individual subjects

Subjects can leave the study at any time for any reason if they wish to do so without any consequences. The investigator can decide to withdraw a subject from the study for urgent medical reasons.

8.4.1 Specific criteria for withdrawal (if applicable)

Not applicable

8.5 Replacement of individual subjects after withdrawal

There will be no replacement of individual subjects after withdrawal.

8.6 Follow-up of subjects withdrawn from treatment

Subjects withdrawn from the study will receive every treatment as usual and all the follow ups and after care as foreseen for every other burn patient.

8.7 Premature termination of the study

No criteria are foreseen for premature termination of the study.

9. SAFETY REPORTING

9.1 Temporary halt for reasons of subject safety

In accordance to section 10, subsection 4, of the WMO, the sponsor will suspend the study if there is sufficient ground that continuation of the study will jeopardise subject health or safety. The sponsor will notify the accredited METC without undue delay of a temporary halt including the reason for such an action. The study will be suspended pending a further positive decision by the accredited METC. The investigator will take care that all subjects are kept informed.

9.2 AEs, SAEs and SUSARs

9.2.1 Adverse events (AEs)

Adverse events are defined as any undesirable experience occurring to a subject during the study. Known adverse events in burn treatment, including both skin grafting techniques, include:

- Post-operative pain
- Post-operative bleeding
- Wound infection
- Impaired healing of donorsite
- Reduced rate of epithelisation of skin graft

The aim of this study is to compare both skin grafting techniques. Therefore these adverse events are not related to the study and will not be reported as adverse events. They will be monitored and listed as secondary outcome parameters.

9.2.2 Serious adverse events (SAEs)

A serious adverse event is any untoward medical occurrence or effect that

- results in death;
- is life threatening (at the time of the event);
- requires or prolongs inpatient (unexpected) hospitalization ;

An elective hospital admission will not be considered as a serious adverse event.

The sponsor will report these SAEs through the web portal *ToetsingOnline* to the accredited METC that approved the protocol, within 7 days of first knowledge for SAEs that result in death or are life threatening followed by a period of maximum of 8 days to complete the initial preliminary report. All other SAEs will be reported within a period of maximum 15 days after the sponsor has first knowledge of the serious adverse events.

Serious adverse events that can occur after surgical treatment in general are defined as; any untoward medical occurrence or effect that results in:

- Pneumonia;
- Urinary tract infections;
- Sepsis;
- Pulmonary embolism;
- Re-operation

9.2.3 Since the aim of this study is to compare both skin grafting techniques, these serious adverse events are probably not related to the study. However, these SAEs are serious by its nature and therefore will be reported yearly to the METc through a line listing. Suspected unexpected serious adverse reactions (SUSARs)

There are no SUSARs expected during this study. No additional risks on adverse events are expected, seen both skin grafting techniques are already frequently used in daily practice of (burn) wounds. In case there is unexpected events which meets the criteria of a SUSAR the sponsor will report expedited the following SUSARs through the web portal *ToetsingOnline* to the METC <*reporting via webportalToetsingOnline is only applicable for investigator initiated studies*>:

- SUSARs that have arisen in the clinical trial that was assessed by the METC;
- SUSARs that have arisen in other clinical trials of the same sponsor and with the same medicinal product, and that could have consequences for the safety of the subjects involved in the clinical trial that was assessed by the METC.

The remaining SUSARs are recorded in an overview list (line-listing) that will be submitted once every half year to the METC. This line-listing provides an overview of all SUSARs from the study medicine, accompanied by a brief report highlighting the main points of concern. The expedited reporting of SUSARs through the web portal Eudravigilance or ToetsingOnline is sufficient as notification to the competent authority.

The sponsor will report expedited all SUSARs to the competent authorities in other Member States, according to the requirements of the Member States.

The expedited reporting will occur not later than 15 days after the sponsor has first knowledge of the adverse reactions. For fatal or life threatening cases the term will be maximal 7 days for a preliminary report with another 8 days for completion of the report.
is will be reported

9.3 Annual safety report

In addition to the expedited reporting of SUSARs, the sponsor will submit, once a year throughout the clinical trial, a safety report to the accredited METC, competent authority, and competent authorities of the concerned Member States.

This safety report consists of:

- a list of all suspected (unexpected or expected) serious adverse reactions, along with an aggregated summary table of all reported serious adverse reactions, ordered by organ system, per study;
- a report concerning the safety of the subjects, consisting of a complete safety analysis and an evaluation of the balance between the efficacy and the harmfulness of the medicine under investigation.

9.4 Follow-up of adverse events

All AEs will be followed until they have abated, or until a stable situation has been reached. Depending on the event, follow up may require additional tests or medical procedures as indicated, and/or referral to the general physician or a medical specialist.

SAEs need to be reported till end of study within the Netherlands, as defined in the protocol

9.5 [Data Safety Monitoring Board (DSMB) / Safety Committee]

Not applicable.

10. STATISTICAL ANALYSIS

10.1 Primary study parameter(s)

Scar quality 12 months post-surgery assessed by the observer score of the POSAS scale will be tested for normality before univariate analyses will be performed. If the data are normally distributed, a paired student's t-test will be used for comparison of the data. Otherwise the Wilcoxon signed-rank test will be applied.

10.2 Secondary study parameter(s)

All continuous data will be assessed with a paired student's t-test, or when data are nog normally distributed with a Wilcoxon signed-rank test.

10.3 Other study parameters

Descriptive statistics, such as means and frequencies.

10.4 Interim analysis (if applicable)

Not applicable.

11. ETHICAL CONSIDERATIONS

11.1 Regulation statement

This study will be conducted according to the principles of the Declaration of Helsinki (Fortaleza, October 2013) and in accordance with the Medical Research Involving Human Subjects Act (WMO) and the valid Dutch laws.

11.2 Recruitment and consent

Patients will be recruited in the Dutch burn centre in Beverwijk and the Belgium burn centre in Ghent. The attending doctor will determine if patient fulfils all inclusion criteria. If so, the attending doctor will discuss the content of the study, intensity for the patients and total study time with patient or their legal representative. If patient/legal representatives is interested, the attending doctor will ask for him/her permission to receive more information about the study during an additional conversation with a member of the research team. During this additional conversation a member of the research team will hand over the patient information form and the permission form of the study. Patients/legal representatives have at least 24 hours to consider their decision. Thereafter, patients/legal representatives are asked by a member of the research team to sign the permission form (informed consent). Both researcher and patient/legal representatives have to sign the informed consent. For questions they can contact the local principal investigator or the independent physician.

11.3 Objection by minors or incapacitated subjects (if applicable)

The code of conduct minors is not applicable.

11.4 Benefits and risks assessment, group relatedness

Patients experience no additional risks since skin grafting according to micrografting technique and meshed skin graft technique are both standard treatments.
As the POSAS, quality of life- and mobility surveys, Cutometer and Dermaspectometer are non-invasive measurements requiring limited registration time the total duration of all measurements is estimated at 60 minutes per subject. No additional risks are to be expected.

11.5 Compensation for injury

The sponsor/investigator has a liability insurance which is in accordance with article 7 of the WMO.

The sponsor (also) has an insurance which is in accordance with the legal requirements in the Netherlands (Article 7 WMO). This insurance provides cover for damage to research subjects through injury or death caused by the study.
The insurance applies to the damage that becomes apparent during the study or within 4 years after the end of the study.

11.6 Incentives (if applicable)

Participants participating in this study receive a full compensation of the parking costs of the two follow-up appointments at the outpatient clinic. Parking fee comes down to 1,10 euro per hour. Travel costs will not be compensated, seen study measurements are performed only during regular follow-up appointments and therefore no additional travel costs are made.

12. ADMINISTRATIVE ASPECTS, MONITORING AND PUBLICATION

12.1 Handling and storage of data and documents

Data will be documented in an online CRF (Castor EDC, Ciwit BV, Amsterdam, the Netherlands). Castor EDC has been audited on good clinical practice (GCP) compliance by Profess Medical Consultancy and has obtained a GCP compliance certificate. After enrolment, the patients' data will be coded by:

- A letter B (Beverwijk) or G (Ghent) for the burn centre;
- A study code: MvsM;
- Number of patients in the study;

E.g.: B-MvsM-001

The key to the codes will be available to local coordinating investigator in the Red Cross Hospital, Beverwijk. The encoded data from each centre will be exported as a CSV-file to the study folder at the secured server of the Red Cross Hospital, enabling statistical analyses in SPSS for Windows. Both teams of investigators in burncentres in Beverwijk and Ghent have access to the encoded data from both centres.

All source data, including signed informed consent forms, are stored locally on a secured server. Access to the server is limited to the local investigators, coordinating investigators, and if necessary, to the members of the medical ethics committee and the health care inspectorate (IGJ). The handling of personal data will be in compliance with the AVG/GDPR. Data will be stored for fifteen years.

The data transfers between University Hospital Ghent and the Red Cross Hospital will be covered by Data Transfer Agreements, in accordance with the AVG/GDPR.

12.2 Monitoring and Quality Assurance

Monitoring will not be performed for this study.

12.3 Amendments

Amendments are changes made to the research after a favourable opinion by the accredited METC has been given. All amendments will be notified to the METC that gave a favourable opinion. The start of the study will be notified to the METc.

12.4 Annual progress report

The sponsor/investigator will submit a summary of the progress of the trial to the accredited METC once a year. Information will be provided on the date of inclusion of the first subject, numbers of subjects included and numbers of subjects that have completed the trial, serious adverse events/ serious adverse reactions, other problems, and amendments.

12.5 Temporary halt and (prematurely) end of study report

The investigator/sponsor will notify the accredited METC of the end of the study within a period of 8 weeks. The end of the study is defined as the last patient's last visit.

The sponsor will notify the METC immediately of a temporary halt of the study, including the reason of such an action. In case the study is ended prematurely, the sponsor will notify the accredited METC within 15 days, including the reasons for the premature termination. Within one year after the end of the study, the investigator/sponsor will submit a final study report with the results of the study, including any publications/abstracts of the study, to the accredited METC.

12.6 Public disclosure and publication policy

The funders had no role in study design, data collection and analysis, decision to publish, or preparation of the manuscript.

13. STRUCTURED RISK ANALYSIS

13.1 Potential issues of concern

Not applicable.

13.2 Synthesis

Patients experience no additional risks since skin grafting according to micrografting technique and meshed skin graft technique are both standard treatments.

14. REFERENCES

1. Surgical procedures of Meek technique https://ozmedix.com.au/humeca/meek/
2. Zermani, R.G., Zarabini, A. and Trivisonno, A., *Micrografting in the treatment of severely burned patients*. Journal of the International Society for Burn Injuries, 1997. 23(7-8): p. 604-7.
3. Zhen, L. and Halim, A., *Superior long term functional and scar outcome of Meek micrografting compared to conventional split thickness skin grafting in the management of burns*. Burns, 2019. 45(6): p. 1386-1400.
4. Lumenta, D. et al., *Comparison of meshed versus MEEK micrografted skin expansion rate: claimed, achieved, and polled results*. Plastic and reconstructive surgery, 2011. 128(1): p. 40e-1e.
5. Vandeput, J. et al. *A review of skin meshers.* Burns, 1995; 21: 364-70
6. Henderson, J., Arya, R and Gillespie, P. *Skin graft meshing, over-meshing and cross-meshing*. International Journal of Surgery. 10 (2012) 547-550
7. Quintero, E., Machado, J., Robles, R. *Meek micrografting history, indications, technique, physiology and experience: a review article*. 2018, Journal of Wound care. 1;27 :S12-S18
8. Fortaleza, Brazil, 2013, *Declaration of Helsinki*
